# Supplementary material for: Laser communications system with drones as relay medium for healthcare applications
Source: PeerJ Comput Sci. 2024 Feb 7;10:e1759. doi: 10.7717/peerj-cs.1759 (PMC10909153; doi:10.7717/peerj-cs.1759)
Supplement: Supplemental Information 3 [file peerj-cs-10-1759-s003.docx]

| **Lighting Condition** | **Successful Transmission Rate (%)** |
| --- | --- |
| Dim Light | 1 |
| Dim Light | 1 |
| Dim Light | 1 |
| Bright Light | 1 |
| Bright Light | 0.9 |
| Bright Light | 0.8 |
| Fluctuating Light | 0.6 |
| Fluctuating Light | 0.8 |
| Fluctuating Light | 0.8 |
