# Supplementary material for: Laser communications system with drones as relay medium for healthcare applications
Source: PeerJ Comput Sci. 2024 Feb 7;10:e1759. doi: 10.7717/peerj-cs.1759 (PMC10909153; doi:10.7717/peerj-cs.1759)
Supplement: Supplemental Information 4 [file peerj-cs-10-1759-s004.docx]

| **Distance (m)** | **Successful Transmission Rate (%)** |
| --- | --- |
| 0.5m | 1 |
| 1m | 1 |
| 1.5m | 1 |
| 2m | 1 |
| 2.5m | 0.8 |
| 3m | 0.8 |
| 3.5m | 0.6 |
| 4m | 0.3 |
| 4.5m | 0.3 |
| 5m | 0 |
