# Supplementary material for: Laser communications system with drones as relay medium for healthcare applications
Source: PeerJ Comput Sci. 2024 Feb 7;10:e1759. doi: 10.7717/peerj-cs.1759 (PMC10909153; doi:10.7717/peerj-cs.1759)
Supplement: Supplemental Information 5 [file peerj-cs-10-1759-s005.docx]

| **Packet Size** | **Dim** | **Bright** | **Fluctuating** |
| --- | --- | --- | --- |
| Small Packets | 1 | 0.95 | 0.88 |
| Medium Packets | 0.97 | 0.9 | 0.82 |
| Large Packets | 0.97 | 0.85 | 0.76 |
